# Coming out ahead: the cost effectiveness of external cephalic version using spinal anesthesia

**DOI:** 10.1186/2045-4015-3-6

**Published:** 2014-02-24

**Authors:** James A O’Brien, Eli Y Adashi

**Affiliations:** 1 Women and Infants Hospital of Rhode Island, 101 Dudley Street, Providence, RI 02905, USA; 2 Department of Obstetrics and Gynecology, Warren Alpert Medical School, Brown University, Providence, RI 02905, USA

## Abstract

Breech presentation is encountered in 3 to 4% of term pregnancies and has been a significant driver of the increased rate of cesarean deliveries over the last 4 decades. External cephalic version (ECV) is recommended at term by most professional organizations in an effort to reduce the prospect of cesarean deliveries. The authors propose the use of regional anesthesia to increase efficacy and reduce cost in the care of patients who undergo ECV in an effort to convert a breech presentation to a vertex counterpart. Despite emerging evidence of the advantages, obstacles to more comprehensive implementation of this approach continue to exist, which include patient acceptance, provider experience, and safety concerns. The addition of tocolytics and use of regional anesthesia for secondary ECV efforts have also been considered as options to increase success and reduce cost. This is a commentary on http://www.ijhpr.org/content/3/1/5.

## Commentary

The 1980’s and 90’s had seen a significant decline in the rate of vaginal breech delivery as per observational studies and anecdotal reports. As such, this trend was facilitated by an increasingly litigious environment in many parts of the world. With the publication of the Term Breech Trial in 2000 [1] a further increase has been noted in the prevalence of cesarean delivery for breech presentation, now as high as 98% in some populations [2]. Despite the fact that the methodology and implications of the Term Breech Trial have been questioned and that several professional organizations have since endorsed breech vaginal delivery in properly selected patients, it is unlikely that the preference for cesarean breech delivery will abate any time soon.

Breech presentation constitutes the third most common indication for cesarean delivery and now accounts for approximately 15% of all cesarean sections performed in the US at an estimated annual cost of $1.4B [3]. In an effort to decrease the incidence of cesarean deliveries for breech presentation, professional organizations recommend offering most patients external cephalic version (ECV) at term. Reported success rates for ECV have ranged from 35 to 86%, with an average of 58% [4]. With renewed interest in this technique, considerable research has been devoted to interventions that will improve the success and the patient acceptance of ECV. In addition to these efforts to reduce the overall incidence of cesarean deliveries and their inherent morbidities, it is essential to assess the cost of the relevant interventions to the healthcare system.

In this month’s issue of the *Israel Journal of Health Policy Research*, Weiniger and colleagues evaluate the hospital costs associated with alternative clinical approaches to breech presentation [5]. In this secondary analysis of data from two prior studies (by applying adjusted cost data), the authors conclude that external cephalic version under neuraxial blockade is a cost-effective approach to the challenges presented by the breech presentation. As it stands, the current management options for the breech presentation in the Hassadah-Hebrew University Medical Center, the site of their study, are the same as in most academic centers – perform ECV or proceed to an elective cesarean section. In the study of Weiniger et al., ECV under spinal anesthesia resulted in successful version in 76% of the cases with 84% of the women in question delivering vaginally. These results were estimated to translate into annual cost savings to the Israeli healthcare system of over 20 M NIS ($5.9 M). The authors also correctly point out that healthcare costs differ greatly between countries and that even greater potential savings could be realized in countries wherein manpower costs are higher. The authors also cite the added benefits to the population of improved quality adjusted life years (QALYs) that coincide with a reduction in cesarean delivery rates.

This report concurs with prior studies analyzing costs associated with ECV. Using a computer-based decision model, Tan et al. [6] concluded that from a societal perspective, an ECV trial was cost-effective when compared to a scheduled cesarean section for breech presentation provided the probability of successful ECV was greater than 32%. Gifford et al. [7], in turn, using decision-analysis techniques, demonstrated a decrease in the cesarean delivery rate as well as cost savings for patients delivering after unsuccessful versions by scheduled cesarean delivery versus routinely scheduling a cesarean when a breech presentation is identified at term.

The data cited in this study represents a compilation of two previous publications by the author. The prospective randomized controlled trials in question each revealed significantly increased rates of successful ECV in nulliparous and multiparous populations using spinal anesthesia. The initial study [8], carried out in nulliparous patients, demonstrated successful ECV in 66.7% of patients receiving spinal anesthesia versus 32.4% in untreated counterparts. The study of multiparous patients [9] also showed a significant success with spinal anesthesia (87.1%) versus without such intervention (57.5%). Both studies documented a significant decrease in visual analogue pain scores in patients receiving spinal anesthesia. The findings of these two smaller studies were confirmed in a meta-analysis [10] that included 4 additional randomized controlled trials.

Although the case for increased success and reduced costs for ECV is clear, some concerns and controversy remain. Patient acceptance has not been universal with reported rates of maternal refusal of an ECV attempt ranging from 18 to 76% [11]. In their vignette study Vlemmix et al. [12] showed that expected procedural pain and the success rate thereof were the most important factors influencing patient willingness to undergo ECV. The use of regional anesthesia would seem to satisfy both of these concerns. Provider experience and comfort level may also impact utilization as 4 to 33% of patients suitable for ECV were not offered the procedure [11]. In situations wherein provider reluctance due to inexperience is the case, it may be reasonable and advantageous for institutions to consider offering the services of more experienced providers to perform ECV.

ECV is felt to be a safe procedure. However, safety concerns are still raised in some reports. Although most of the controversy dates back to 1970 when procedure-associated fetal mortality was thought to be about 1%, the following is worth considering. The most frequent complication of ECV is transient fetal heart rate changes that have been reported in 5.7% of cases [13]. Persistent changes resulting in the need for emergency cesarean delivery are seen only in 0.3% of cases. The incidence of placental abruption is 0.12% and the prevalence of feto-maternal transfusion appears to range from 2 to 4%. Opponents of the use of anesthesia for ECV argue that complication rates are higher under these circumstances and feel that this may be related to the potential of applying too much force in these pain-free patients.

Other medications have also been used to enhance the success of ECV. Randomized trials have demonstrated that significant improvement may be expected with most tocolytics with the exception of nifedipine. These medications have been used in conjunction with regional anesthesia in some studies and likely provide a combination of myometrial and abdominal muscular relaxation. The combined use of a tocolytic and regional anesthesia appears to be associated with an increased success rate.

The literature regarding the use of regional anesthesia features studies using spinal, epidural, and combined spinal epidural approaches. Most studies seek to achieve adequate anesthesia to the T6 level which may well optimize the likelihood for successful ECVs. Several authors emphasize the difference in using anesthesia versus analgesic dosing levels as a key to successful ECV as well. Some also favor the use of combined spinal epidural as a means to providing adequate dosing for an emergency cesarean delivery in the event of a non-resolving fetal bradycardia.

Many authors have tried to define predictors of a successful ECV with little consistency. In a comprehensive meta-analysis [11] involving over 10,000 women, multiparity, non-engagement of the breech, a relaxed uterus, a palpable fetal head, and maternal weight less than 65 kg were shown to be valid positive prognostic indicators.

Smaller studies have assessed alternative approaches using neuraxial anesthesia as a secondary option after an unsuccessful attempted version without anesthesia. Rozenberg et al. [14] resorting to epidural anesthesia reported a 39.7% success rate for ECV after an unsuccessful initial attempt with a beta-mimetic alone. Their overall success rate was 72.8%. This approach may well have the potential to yield additional cost savings.

Successful ECV does not guarantee a vaginal delivery. Spontaneous reversion to the breech presentation can occur in approximately 4% of cases. More significantly, cesarean delivery is more than twice as likely in patients after ECV than in control patients with cephalic presentations [15]. The main indications for the cesarean deliveries in question are dystocia and fetal intolerance of labor. Although several hypotheses have been advanced for these findings, the reasons remain unclear. Kabiri et al. [16] observed in a retrospective cohort study that the risk of cesarean delivery was significantly decreased when delivery occurred more than 96 hours after successful ECV in both nulliparous and multiparous patients.

With cesarean section currently considered as the only delivery option for breech presentation in many settings, efforts to optimize successful external cephalic version will likely assume increased significance. It follows that institutions should consider strategies to gain better patient and provider acceptance and enhance the rates of success. The use of tocolytics and regional anesthesia has been consistently shown to increase ECV success. The study by Weiniger and colleagues demonstrates the additional benefit of decreased costs with this therapeutic strategy.

This is a commentary on Weiniger CF, Spencer PS, Weiss Y, Ginsberg G, Ezra Y. Secondary data analysis of hospital costs for external cephalic version under neuraxial blockade to reduce cesarean delivery for breech presentation at term. Israel Journal of Health Policy Research 2014;3:5.

## Competing interests

The authors declare that they have no competing interests.

## Authors’ information

James A. O’Brien MD, FACOG is the Medical Director of Inpatient Obstetrics at Women and Infants Hospital of Rhode Island. He is also an Assistant Professor of Obstetrics and Gynecology (Clinical) at the Warren Alpert Medical School of Brown University.

Eli Y. Adashi MD, MS, CPE, FACOG is Professor of Medical Science at Warren Alpert Medical School of Brown University.

## Commentary on

Secondary data analysis of hospital costs for external cephalic version under neuraxial blockade to reduce cesarean delivery for breech presentation at term: CF Weiniger MB ChB, PS Spencer PhD, Y Weiss MD MPH, G Ginsberg DrPH, Y Ezra MD.
